# Joint attention and exogenous attention allocation during mother-infant interaction at 12 months associate with 24-month vocabulary composition

**DOI:** 10.3389/fpsyg.2025.1516587

**Published:** 2025-04-17

**Authors:** Elena Capelli, Serena Grumi, Luisa Vercellino, Livio Provenzi

**Affiliations:** ^1^Department of Brain and Behavioral Sciences, University of Pavia, Pavia, Italy; ^2^Developmental Psychobiology Lab, IRCCS Mondino Foundation, Pavia, Italy

**Keywords:** distractibility, infancy, joint attention, language, vocabulary composition distractibility, vocabulary composition

## Abstract

**Introduction:**

Early attentional processes are inherently linked with early parent-infant interactions and play a critical role in shaping cognitive and linguistic development. This study explored how specific early attention mechanisms-namely, exogenous attention allocation and joint attention initiation-during mother-infant interactions at 12 months may influence language development at 24 months.

**Methods:**

A sample of 46 typically developing children was observed at 12 months during mother-infant interactions obtained through remote videotaping. Quantitative measures of exogenous attention allocation to external auditory stimuli and joint attention initiation by the infant were obtained through micro-analytical coding. Language outcomes were assessed at 24 months, with a focus on vocabulary composition (i.e., percentage of predicates).

**Results:**

Findings showed significant negative associations between early life exogenous attention allocation and later vocabulary composition (i.e., predicate percentage). This association was modulated by joint attention initiation: infants displaying lower levels of joint attention initiation showed a negative association between exogenous attention allocation and language development.

**Discussion:**

The findings are suggestive of a complex relationship among different forms of early attention skills and language development in the first 2 years of life.

## Introduction

The transition to the third year of life represents a pivotal turning point for language development. Children's vocabulary tends to experience a significant increase at the end of the second year of life and many children start to combine different words (Rantalainen et al., 2021). This phase is often accompanied by a substantial increase in predicates production. Nonetheless, this developmental milestone contributes to individual differences that are only partly explained. In the present study, we explored how early forms of attention at 12 months (i.e., exogenous attention allocation and joint attention initiation) might modulate individual differences in 24 months language development in typically developing children.

Children's vocabulary shows a dramatic increase in the second year of life, as infants go from learning their first words around their first birthday to an average of 300 words by their second birthday, with significant individual variability (Frank et al., 2017). Moreover, a common pattern can be found in the qualitative composition of this first vocabulary. Namely, in the very first phases of language development a larger proportion of words learned are related to first social interaction and everyday life routines whereas as the vocabulary grows a larger proportion of words learned are object names and subsequently, as combinatory abilities emerge, action (such as verbs and adjectives) and function words tend to augment. This pattern has been reported in different languages (Caselli et al., 1999; Choi and Gopnik, 1995; Maital et al., 2000; Tardif et al., 1999). A restricted percentage of predicates has also been proposed as an early marker of language delay in Italian speaking children (Camaioni and Longobardi, 1995; D'Odorico et al., 2001) and the number of verbs that children produce at age two has been shown to be a better predictor of later grammatical skills than the number of nouns (Hadley et al., 2016). Nevertheless, limited research as focused on predictors of vocabulary composition or on potential mechanisms of predicates learning (Horvath et al., 2021; Horvath and Arunachalam, 2019).

Children learn language in environments that are both rich with information and yet ambiguous, where every label can have multiple possible referents (Raz et al., 2019). We know that the parents' ability to provide labels contingently with the child's attentional focus to a specific object can support learning (Suanda et al., 2016; Yurovsky et al., 2013) and early interventions focused on parent-infant interactions capitalize on this core assumption (Buschmann et al., 2015; Fong et al., 2012; Roberts and Kaiser, 2015). Consistent with expectations, numerous studies have documented links between children's attentional abilities and their language development. This association appears to hold true across various attentional skills (Salley et al., 2013).

Attention allocation is guided by endogenous mechanisms entailing the ability to actively maintaining focus on a stimulus (sustained attention) and exogenous mechanisms leading to shifts to environmental stimuli or distractors (distractibility) (Colombo, 2001; Colombo and Cheatham, 2006). Endogenous attention allocation develops early in the first year of life but continues to improve in the second half of the first year, enhancing visual engagement, and attention allocation control (Lansink et al., 2000; Reynolds and Romano, 2016).

Higher levels of sustained attention in 9-month-olds have been linked with better language outcomes 6 months later (Yu et al., 2019). Consistently, 12-month-old infants showing frequent exogenous attention allocation to external stimuli have been described as achieving less optimal language development 6 months later (Testa et al., 2023). Notably, prior investigations in this domain have been confined to controlled laboratory environments. Consequently, the influence of early exogenous attention allocation within naturalistic, interactive social contexts involving infants and their caregivers remains largely unexplored. To bridge this knowledge gap, future research should elucidate how this facet of attention relates to subsequent language development when assessed in ecologically valid settings.

Furthermore, between 9 and 12 months, infants show significant advancements in social attention development, including joint attention engagement i.e., the ability to share their attentional focus on external stimuli with the caregivers (Bradley, 2023). In this developmental window infants are sensitive to other's gaze direction and show gradual increases in gaze- and point-following, responding to joint attention cues (Tang et al., 2024) and further expanding their ability to initiate joint attention, using their own gaze and other social cues to intentionally share attention with others (Stephenson et al., 2021). Links between joint attention and language development have been described, too (Tomasello and Todd, 1983) and social-pragmatic cues remain important sources of information about word meanings even for adults. For instance, infants learn to use the adult's gaze to acquire new words using this cue to develop word-to-world mappings (Hollich et al., 2000). Coherently, significant associations have been reported between language development and child ability to both respond to joint-attention (Delgado et al., 2002; Morales et al., 2000) and initiate joint attention by sending specific bids in the shape of gaze triangulation and early communicative behaviors (e.g., pointing; Bavin et al., 2008; Brooks and Meltzoff, 2008; McGillion et al., 2017). As previously highlighted, evidence of a link between early life exogenous attention allocation and later cognitive and language development is present in the literature (Colombo and Mitchell, 2009; Hendry et al., 2019; Wass, 2015). Although multiple studies have shown that children are active agents in their environment and their abilities and motivations greatly impact learning mechanisms (Thelen, 1995), previous research has only partly focused on the role played by children's initiation of joint attention in influencing later language outcomes.

Underlying causes of individual variability in language development are still partly unknown, as many studies on language development predictors only explain a small portion of the difference in vocabulary size (Reilly et al., 2018). Moreover, studies often investigate the main effect of potential predictors and don't account for interaction effects between predictors; conversely recent studies have highlighted the presence of complex and cumulative effects of risk factors in later development (Eadie et al., 2022). For instance, the presence of multiple risk factors has been shown to increase the probability of language delays in the general population (Hayiou-Thomas et al., 2021). However, limited research has investigated potential interactive effects between attention related measures and later language development. This dearth of evidence is regrettable considering that language development (as development in general) is shaped by diverse, interconnected, interdependent mechanisms, and its nature should prompt us to embrace complexity (D'souza et al., 2017). These dynamic interconnected effects are even more relevant in the case of attentional risk and protective factors as attentional mechanisms can both support one another (e.g., Fisher, 2019) or act in competition (e.g., Lee and Schumacher, 2024). For instance, limited studies have investigated the potential moderating effect of joint attention on the link between early individual cognitive differences and later language development (e.g., Canfield and Saudino, 2016; Salley et al., 2013) with contrasting results. For instance, recent studies have shown that infant sustained attention in the context of joint attention, but not joint attention itself, seem to be a stronger predictor of later vocabulary size, suggesting that joint attention may not just co-occur with infant sustained attention but may play a key supportive role (Yu et al., 2019). As such good joint attention skills may increase the protective effect of sustained attention or reduce the reported negative effect of frequent exogenous attention allocation on later development. On the other hand, research has shown that individual susceptibility to environmental cues in early life (i.e., environmental sensitivity—Bahrick et al., 2018; Moyano et al., 2023) can make individuals more malleable to both its positive and negative influences, essentially making them more responsive to both risk and protective factors (Greven et al., 2019). As such exogenous attention allocation—framed as an index of environmental sensitivity i.e., heightened openness to the environment—may act as a mediator in word learning enhancing or reducing the reported positive effects of joint attention engagement.

Thus, the goals of the present study were: (a) to investigate associations between 12-month exogenous attention allocation (to social auditory stimuli), joint attention initiation—measured in real-life social settings—and language development at 24 months assessing both vocabulary dimension and composition; (b) to explore the presence of interactive/modulating effects between 12-month infants' exogenous attention allocation and initiation of joint attention in predicting later language development.

## Methods

### Participants

Forty-six children and their families participated in the study as part of a spin-off follow-up of a longitudinal study on the association between early environmental exposures and child cognitive and emotional developmental trajectories—*masked for peer review—*. Families were recruited at birth from 10 local Neonatal Units in—*masked for peer review—*. All children were born at term, from healthy pregnancies, and had no neurological or sensory deficit diagnoses. Two waves of data collection have been selected for the purposes of the present study: 12-month-age (T1) and 24-month-age (T2). The study was approved by the—*masked for peer review—*Ethics Committees. All families provided informed consent prior to participating in the study.

### Procedures and measures

#### Observational measures of attention

At infants' 12-month-age (a relevant developmental window where the first word production typically emerges), mothers and infants participated to a video-recorded face-to-face interaction via teleconferencing. Mothers were instructed to position their child in a highchair and sit a short distance away to facilitate interaction. They were advised not to use toys or pacifiers. The device used for the connection was to be set horizontally, providing a clear view of both partners' torsos and faces, with the screen set to blank. The procedure comprised five consecutive episodes (see *masked reference for blind review*). Initially, they engaged in unstructured face-to-face interaction for 2 min. Next, researchers introduced a series of three sounds, presented one at a time. These sounds included both socially meaningful utterances like “hello” and “how nice,” as well as neutral sounds like flowing water and a mixer. Each sound had the same duration. While the sounds played (30 s), mothers were instructed to maintain neutral expressions and avoid talking to their children. The request made to the caregiver aimed to maintain the child's motivational state, encouraging them to re-engage the adult interactive partner that has stopped the interaction abruptly. This approach aligns with previous studies on infants' responses to brief interruptions and manipulations of face-to-face interactions, which highlight the importance of sustaining engagement in social exchanges (Mesman et al., 2009; Provenzi et al., 2016). After each sound exposure, a play resumption period of 30 s allowed for free interaction again. This exposure-reprise sequence was repeated four times, with the order of sounds varied between mother-child pairs to control for order effects. By employing a blank screen on the device used for the connection, the study focused solely on how mothers and children responded to the auditory stimuli within the context of their ongoing interaction. Consistent with the goals of the present study, socially meaningful exposure episodes have been selected for data analysis.

#### Evaluation of vocabulary development

The mothers filled in an Italian adaptation of the McArthur Bates Communication Development Inventory (CDI; Fenson et al., 1993), the “*Il primo vocabolario del bambino*” Words and Action/Gestures short form (Caselli et al., 2015). The questionnaire is normed on a sample of typically developing Italian children between the ages of 8 and 30 months. The Words and Action/Gestures short form assesses the onset of communication skills, between 8 and 24 months of the infants' life. The first section of the questionnaire comprise a list of 100 words; the parent is asked to indicate if the child understands and/or produces each word. Qualitative vocabulary composition was obtained for four major word categories based on operational procedures provided by Bates et al. (1994): (a) Social words—containing sound effects and animal sounds, names for people, and games and routines (b) Nouns—including semantic categories with a clear naming function: animals, vehicles, toys, food and drinks, clothing, body parts, household objects, and furniture and house rooms (c) Predicates—containing two semantic categories: action words (verbs) and descriptive words (adjectives) (d) Closed-class words—including pronouns, prepositions, question words, quantifiers and articles, and connecting words.

### Data reduction

#### Observational measures of attention

Exposure episodes were micro-analytically coded. *Exogenous attention allocation* (*EAA*) was coded as the proportion of time infant's face and/or gaze was clearly directed toward the auditory stimulus source, as an index of time spent looking at the distracting stimulus. Conversely, the proportion of time infant's face and/or gaze was directed to the mother was coded as *Social Attention to mother* (SAM). *Joint attention initiation* (*JAI*) was coded as the frequency of occurrence of the following sequence of three infant gaze behaviors within 2 s of each other: (1) orienting to the auditory stimulus source; (2) orienting to the mother; (3) orienting back to the auditory stimulus source. This allowed us to highlight instances in which the infants used their gaze to signal an attentional shift, triangulating their gaze between the stimulus and the interactive partner. A subset of randomly selected videos (10%) was independently coded by two coders and the inter-rater agreement was above 95% for all tested dyads.

#### Evaluation of vocabulary development

From the CDI questionnaire we computed the child's vocabulary size as the number of words the parent reported the child to use (CDI production). Composition percentages were computed for the four word-categories: social, nouns, predicates, and closed class words.

#### Statistical power and sample size estimation

Sample size was estimated based on the final regression model (planned with three predictors and one interaction effect) and setting parameters as follows: *f*^2^ = 0.30, α = 0.05, β = 0.20. A total sample size of *n* = 45 was estimated.

### Plan of analyses

Associations between EAA, SAM, JAI, and later language measures were tested via Pearson bivariate correlations. Correction for multiple comparisons (Benjamini-Hochberg false discovery rate procedure) was applied. A regression model was used to assess potential significant interactive effects between EAA and JAI on later language outcomes for all attention-language associations that survived multiple comparison correction. Regression analyses will include control for the potential confounding effect of infants' sex. All analyses were conducted with Jamovi 2.5 for Windows 11.

## Results

### Characteristics of the sample

The socio-demographic sample description is reported in Table 1. Of the 46 children, 21 were females (46%). Children and their caregiver participated in two waves of data collection: time-point (1) around 12-month-age (M = 12.40; SD = 0.34) and time-point (2) around 24-month-age (M = 24.15; SD = 0.67). Descriptive analyses of variables measured at the 12- and 24-months data collection points are presented in Table 2.

**Table 1:** Socio-demographic descriptive statistics.

**Variables**	**Mean**	**SD**	**Minimum**	**Maximum**
Gestational age (weeks)	39.70	1.12	37	42
Birth weight (grams)	3,301	369	2,580	4,260
Maternal age (years)	33.30	4.08	26	43
Paternal age (years)	34.80	5.43	20	51
Maternal education (school years)	15.90	2.79	8	21
Paternal education (school years)	14.50	3.31	8	21

**Table 2:** Descriptive statistics for T1 (12 months) and T2 (24 months) data.

**Variables**	**Mean**	**SD**	**Minimum**	**Maximum**
**T1—Observational measures of attention**				
EAA exposure (% time)	24.03	8.68	5.00	46.50
SAM exposure (% time)	11.05	6.10	0.00	25.00
JAI (frequency)	2.43	1.89	0.00	8.00
**T2—Evaluation of vocabulary development**				
CDI production (frequency)	67.50	29.23	3.00	100.00
Social words (%)	19.90	11.67	6.12	66.70
Nouns (%)	51.40	11.80	0.00	93.90
Predicates (%)	15.70	7.79	0.00	24.20
Closed class (%)	13.00	4.92	0.00	33.30

EAA, exogenous attention allocation to sound; SAM, social attention to mother; JAI, joint attention initiation; CDI, McArthur Bates communication development inventory.

### Vocabulary composition

**Figure 1:**
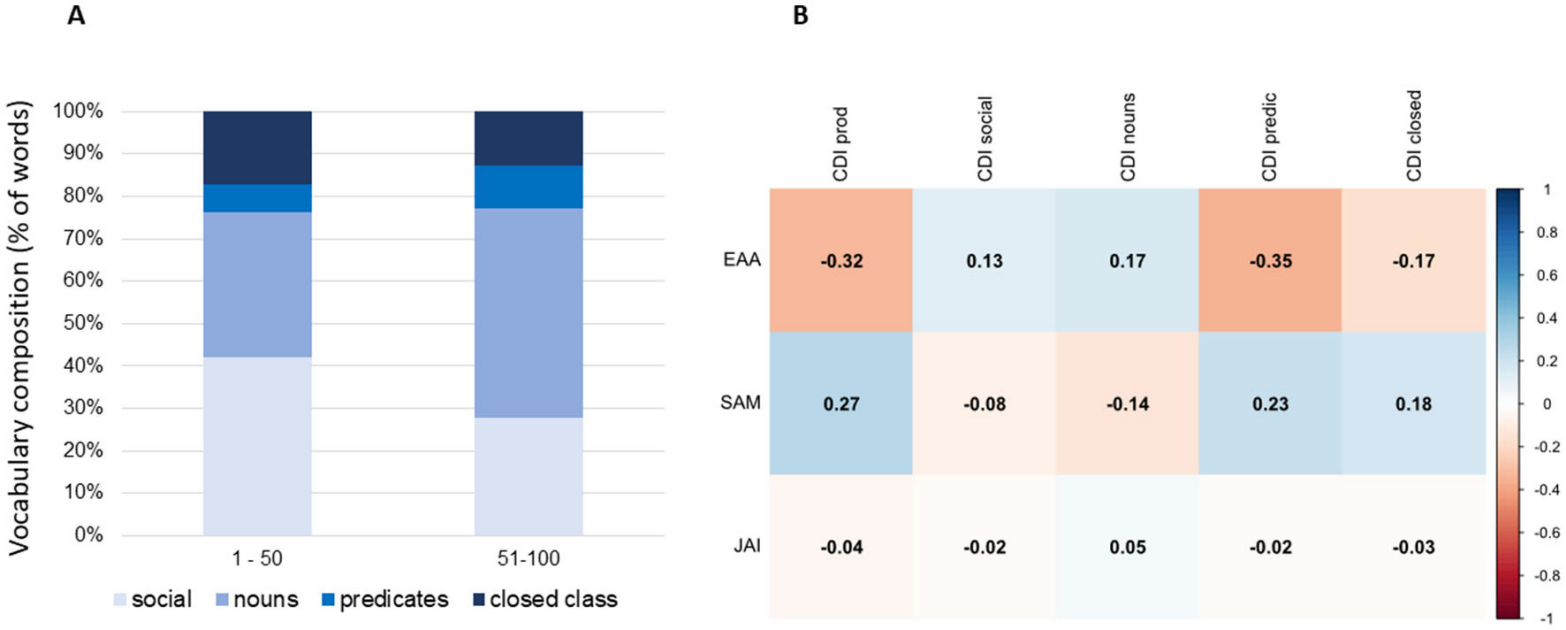
**(A)** Twenty-four months vocabulary distribution (social, nouns, predicates, and closed class words) in two sub-groups of children split by vocabulary size (lower 1–50 words, *n* = 15 vs. higher 50–100 words, *n* = 31); **(B)** Correlation heatmap reporting Pearson's *r* values for the associations between 12-month behavioral measures and 24-month vocabulary; EAA, exogenous attention allocation; SAM, social attention to mother; JAI, joint attention initiation; CDI, McArthur Bates communication development inventory.

Looking at our sample vocabulary composition, we observed significant positive associations between the total vocabulary size (number of words produced) and percentage of nouns [Pearson's *r*_(44)_ = 0.350, *p* = 0.017] and predicates [Pearson's *r*_(44)_ = 0.878, *p* < 0.001]. Conversely negative associations emerged between vocabulary size and percentage of social words [Pearson's *r*_(44)_ = −0.812, *p* < 0.001] and closed class words [Pearson's *r*_(44)_ = −0.303, *p* = 0.041]. This trend was confirmed observing the composition distributions (see Figure 1A) in 2 sub-groups of children split by vocabulary size (lower 1–50 words *n* = 15 vs. higher 50–100 words *n* = 31).

### Attention skills at 12 months and vocabulary development at 24 months

Significant negative correlations emerged for the percentage of time that the infant spent looking toward the social sound source (EAA) during the exposure episode with overall vocabulary size [Pearson's *r*_(44)_ = −0.323, *p* = 0.028] and percentage of predicates [Pearson's *r*_(44)_ = −0.342, *p* = 0.020; Figure 1B]. Only the latter correlation test survived correction for multiple comparisons (Supplementary material S1). The complete correlation table for all measures collected is presented in Supplementary material S2.

**Figure 2:**
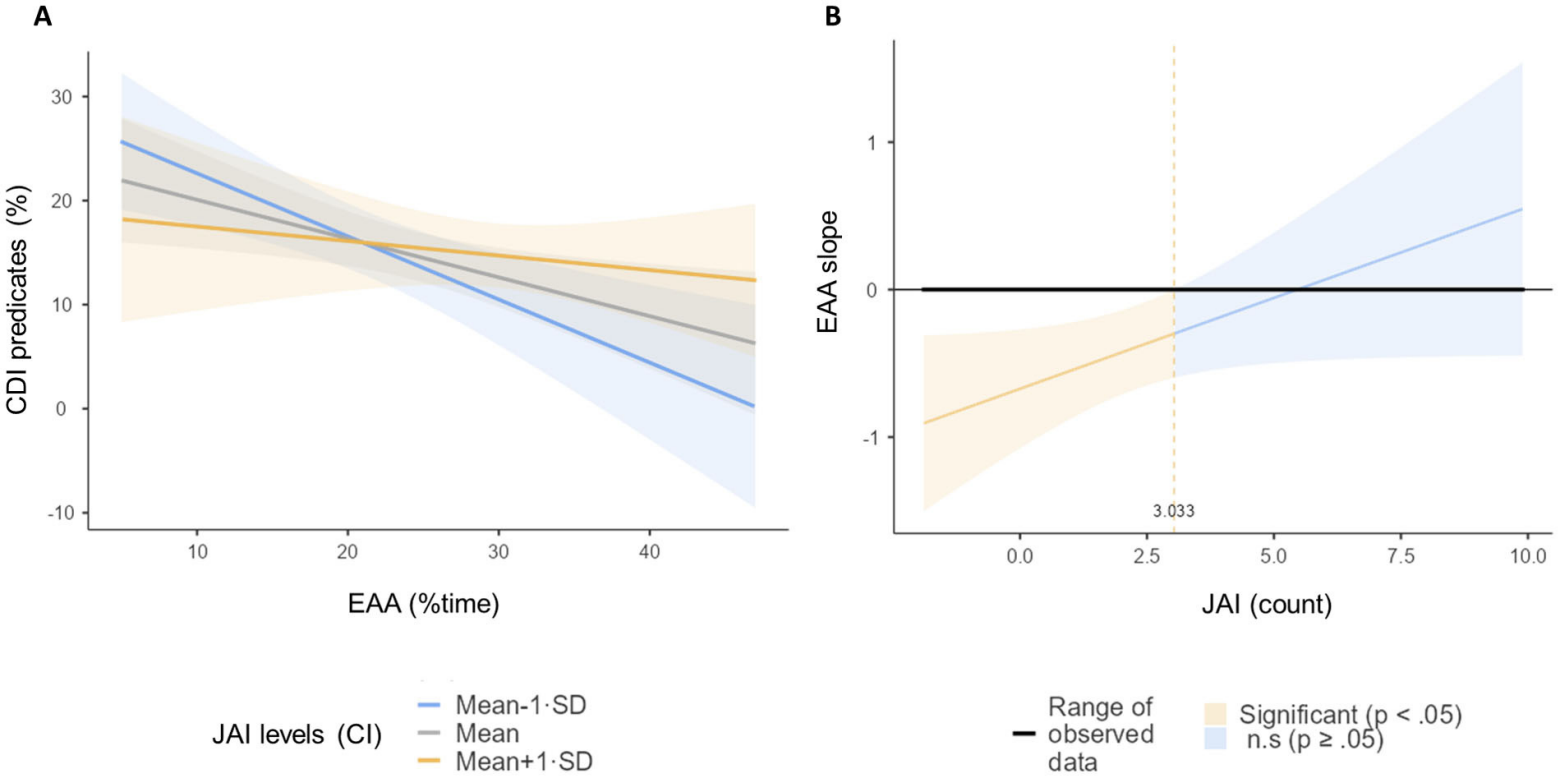
**(A)** Regression slopes for EAA (exogenous attention allocation) during exposure – CDI (Communication Development Inventory) predicates % association at three different Triangulation values (-1SD, mean, +1SD) **(B)** and Johnson-Neyman graph for interaction inspection.

A regression model investigating effects of EAA and JAI abilities on later language (controlling for potential confounding effects of sex) was tested with percentage of predicates as the dependent variable and assessing interaction effects between EAA and JAI (mean centered). The model was statistically significant *F*_(4, 41)_ = 3.12, *p* = 0.025 and explained 23.3% of the variance. The results highlight a significant negative effect of EAA [β = −0.416, *t*_(44)_ = −2.65, *p* = 0.011] with no significant effects of JAI [β = 0.089, *t*_(44)_ = 0.537, *p* = 0.594] and sex [β = −0.287, *t*_(44)_ = −1.05, *p* = 0.299]. Furthermore, a trend for a significant interaction effect between EAA and JAI emerged [β = 0.260, *t*_(43)_ = 1.99, *p* = 0.054]. Interaction was inspected with simple effects *post-hoc* analysis (Figure 2A) and significant slopes further specified with the Johnson-Neyman procedure (Figure 2B). In infants exhibiting greater JAI the negative association between EAA and percentage of predicates was weaker (Figure 2). Simple effects *post-hoc* analyses confirms this observation: the regression slopes for −1 SD, *F*_(1, 41)_ = 11.71, *p* < 0.001, and mean levels of JAI, *F*_(1, 41)_ = 7.01, *p* = 0.011, showed statistically significant associations between EAA and predicates percentage, whereas the +1 SD JAI level slope did not, *F*_(1, 41)_ = 0.54, *p* = 0.466. Conversely, simple slopes based on EAA resulted in non-statistically significant regression slopes between JAI and percentage of predicated for mean and low EAA levels [−1 SD, *F*_(1, 41)_ = 0.50, *p* = 0.482; Mean, *F*_(1, 41)_ = 0.29. *p* = 0.594] and a slope showing a trend for a positive association between JAI and percentage of predicates only for higher levels on EAA [+1 SD *F*_(1, 41)_ = 3.98, *p* = 0.053].

## Discussion

The present study aimed at exploring how early forms of attention at 12 months (i.e., exogenous attention allocation and joint attention initiation) might modulate individual differences in 24 months language development (i.e., vocabulary) in typically developing children.

Higher vocabulary size at 24 months—and specifically more advanced vocabulary composition as signaled by the percentage of predicates—was negatively associated with the amount of time infants' exogenous attention allocation was attracted by external social auditory stimuli during the interaction with their primary caregiver. These findings are consistent with studies previously conducted in more experimental settings (Salley et al., 2013; Testa et al., 2023) highlighting negative associations between distractibility and language development. Moreover, the results can add to the current literature on the importance of early attention allocation in impacting later cognitive development at different levels (Hendry et al., 2019). Although orienting to new external stimuli is certainly an important skill, supporting environmental exploration and learning, it can be hypothesized that the role of this ability could be modulated by different contexts (e.g., interactive vs. individual context) and in relation to different developmental outcomes (e.g., socio-cognitive vs. communicative development). Moreover, if we frame exogenous attention allocation as an individual feature potentially related to the concept of environmental sensitivity (Greven et al., 2019), the present results could suggest that higher levels of sensitivity to the environment may expose infants to higher distractibility and be linked to less optimal language outcomes already at 24 months.

Furthermore, interactive effects between exogenous attention allocation and joint attention initiation emerged. On one hand, infants that exhibited less joint attention initiation bids showed a stronger association between such distractibility and language development compared to counterparts that produced higher levels of joint attention initiation. As such, joint attention initiation ability appears to emerge as a potential protective factor in the previously presented developmental association between attention and language. A potential explaining hypothesis for this finding may be that the child's ability to actively bring the adults' attention to a distracting event (i.e., by initiating joint attention episodes) could attenuate the negative effects of distractibility by integrating the distracting event in the interaction. If confirmed, these findings could stress the importance of assessing more complex and interactive effects in early development cascades and the relevant active role of the child on his/her own cognitive development (Thelen, 1995). On the other hand, only infants with higher exogenous attention allocation tended to experience positive associations between joint attention initiation and later language. A potential explaining hypothesis for this finding, could be that being more open and responsive to the environment can increase the positive effect of joint attention initiation episodes—for instance creating more opportunities for concurrent maternal language input—on later early vocabulary acquisition. If confirmed this finding could support evidence on the moderating effects of environmental sensitivity on other risk and protective factors (Greven et al., 2019).

The present study has limitations. Firstly, the sample is relatively small and homogeneous, recruited in an urban middle to high-income area, thus the findings require further replication in larger and more diverse cohorts, ideally including also families from different socio-economic and cultural backgrounds. Nonetheless, consistent with the available literature in Italian speaking children (Caselli et al., 1999; D'Odorico et al., 2001), bigger vocabulary size at 24 months featured a larger predominance of predicates. This suggests that—despite the sample size of our study might be relatively small—the representativeness of language data is preserved. Second, despite its longitudinal nature, the present study is observational and the relationships among variables can only be considered in in terms of associations rather than predictions. Furthermore, the study is focused on the link between early attentional abilities and language; we acknowledge the complex and multi-faceted mechanisms underlying communicative and linguistic development and that other factors may intervene and further contribute to the described association framework. Finally, whereas the 12-month measures have been collected observing mother-infant interactive behaviors, vocabulary was tested via parental questionnaire. The CDI is a widely used tool in language development research; notwithstanding, a combination of parent-reported and direct testing would have allowed a broader assessment of language abilities.

## Conclusions

The present study aimed at testing early attention abilities in an interactive social context and assessing not only direct links but also interaction effects between these early abilities and later language. The results of the present study could thus be relevant in expanding our knowledge of early developmental cascades in language acquisition and in supporting the current literature on potential targets for screening and intervention.

## Data Availability

The raw data supporting the conclusions of this article are available in the Zenodo repository and linked by doi: 10.5281/zenodo.12633606. Further enquiries should be directed to the corresponding author.

## References

[B1] Bahrick L. E. Todd J. T. Soska K. C. (2018). The multisensory attention assessment protocol (MAAP): characterizing individual differences in multisensory attention skills in infants and children and relations with language and cognition. Dev. Psychol. 54, 2207–2225. 10.1037/dev0000594 30359058 PMC6263835

[B2] Bates E. Marchman V. Thal D. Fenson L. Dale P. Reznick J. S.. (1994). Developmental and stylistic variation in the composition of early vocabulary. J. Child Lang. 21, 85–123. 10.1017/S0305000900008680 8006096

[B3] Bavin E. L. Prior M. Reilly S. Bretherton L. Williams J. Eadie P.. (2008). The early language in victoria study: predicting vocabulary at age one and two years from gesture and object use. J. Child Lang. 35, 687–701. 10.1017/S0305000908008726 18588721

[B4] Bradley H. (2023). Qualitative and quantitative measures of joint attention development in the first year of life: a scoping review. Infant Child Dev. 32:e2422. 10.1002/icd.2422 37872965 PMC10588805

[B5] Brooks R. Meltzoff A. N. (2008). Infant gaze following and pointing predict accelerated vocabulary growth through two years of age: a longitudinal, growth curve modeling study. J. Child Lang. 35, 207–220. 10.1017/S030500090700829X 18300435

[B6] Buschmann A. Multhauf B. Hasselhorn M. Pietz J. (2015). Long-term effects of a parent-based language intervention on language outcomes and working memory for late-talking toddlers. J. Early Intervent. 37, 175–189. 10.1177/1053815115609384

[B7] Camaioni L. Longobardi E. (1995). Nature and stability of individual differences in early lexical development of Italian-speaking children. First Lang. 15, 203–218. 10.1177/014272379501504405

[B8] Canfield C. F. Saudino K. J. (2016). The influence of infant characteristics and attention to social cues on early vocabulary. J. Exp. Child Psychol. 150, 112–129. 10.1016/j.jecp.2016.05.005 27280332

[B9] Caselli C. Casadio P. Bates E. (1999). A comparison of the transition from first words to grammar in English and Italian. J. Child Lang. 26, 69–111. 10.1017/S0305000998003687 10217890

[B10] Caselli M. C. Bello A. Rinaldi P. Stefanini S. Pasqualetti P. (2015). Il Primo Vocabolario del Bambino: Gesti, Parole e Frasi. Valori di riferimento fra 8 e 36 mesi delle Forme complete e delle Forme brevi del questionario MacArthur-Bates CDI. Milano: Franco Angeli.

[B11] Choi S. Gopnik A. (1995). Early acquisition of verbs in Korean: a cross-linguistic study. J. Child Lang. 22, 497–529. 10.1017/S0305000900009934 8789512

[B12] Colombo J. (2001). The development of visual attention in infancy. Annu. Rev. Psychol. 52, 337–367. 10.1146/annurev.psych.52.1.337 11148309

[B13] Colombo J. Cheatham C. L. (2006). The emergence and basis of endogenous attention in infancy and early childhood. Adv. Child Dev. Behav. 34, 283–322. 10.1016/S0065-2407(06)80010-8 17120808

[B14] Colombo J. Mitchell D. W. (2009). Infant visual habituation. Neurobiol. Learn. Memory 92, 225–234. 10.1016/j.nlm.2008.06.002 18620070 PMC2758574

[B15] Delgado C. E. F. Mundy P. Crowson M. Markus J. Yale M. Schwartz H. (2002). Responding to joint attention and language development. J. Speech Lang. Hearing Res. 45, 715–719. 10.1044/1092-4388(2002/057) 12199401

[B16] D'Odorico L. Carubbi S. Salerni N. Calvo V. (2001). Vocabulary development in Italian children: a longitudinal evaluation of quantitative and qualitative aspects. J. Child Lang. 28, 351–372. 10.1017/S0305000901004676 11449943

[B17] D'souza D. D'souza H. Karmiloff-Smith A. (2017). Precursors to language development in typically and atypically developing infants and toddlers: the importance of embracing complexity. J. Child Lang. 44, 591–627. 10.1017/S030500091700006X 28393740

[B18] Eadie P. Levickis P. Mckean C. Westrupp E. Bavin E. Ware R.. (2022). Developing preschool language surveillance models—cumulative and clustering patterns of early life factors in the early language in Victoria Study Cohort. Front. Pediatr. 10:826817. 10.3389/fped.2022.826817 35186809 PMC8854765

[B19] Fenson L. Dale P. S. Reznick J. S. Thal D. Bates E. Hartung J. P. (1993). The MacArthur-Bates Communicative Development Inventories: User's Guide and Technical Manual. New York, NY: Brookes.

[B20] Fisher A. V. (2019). Selective sustained attention: a developmental foundation for cognition. Curr. Opin. Psychol. 29, 248–253. 10.1016/j.copsyc.2019.06.002 31284233

[B21] Fong N. W. Y. Ho S. K. Y. So B. J. W. Lian W. B. (2012). Evaluation of the Hanen it takes two to talk intervention programme. Proc. Singapore Healthc. 21, 251–256. 10.1177/201010581202100406

[B22] Frank M. C. Braginsky M. Yurovsky D. Marchman V. A. (2017). Wordbank: an open repository for developmental vocabulary data. J. Child Lang. 44, 677–694. 10.1017/S0305000916000209 27189114

[B23] Greven C. U. Lionetti F. Booth C. Aron E. N. Fox E. Schendan H. E.. (2019). Sensory processing sensitivity in the context of environmental sensitivity: a critical review and development of research agenda. Neurosci. Biobehav. Rev. 98, 287–305. 10.1016/j.neubiorev.2019.01.009 30639671

[B24] Hadley P. A. Rispoli M. Hsua N. Nippold M. Hoffman L. (2016). Toddlers' verb lexicon diversity and grammatical outcomes. Lang. Speech Hearing Serv. Sch. 47, 44–58. 10.1044/2015_LSHSS-15-0018 26803292

[B25] Hayiou-Thomas M. E. Smith-Woolley E. Dale P. S. (2021). Breadth versus depth: cumulative risk model and continuous measure prediction of poor language and reading outcomes at 12. Dev. Sci. 24:e12998. 10.1111/desc.12998 32449284 PMC11475567

[B26] Hendry A. Johnson M. H. Holmboe K. (2019). Early development of visual attention: change, stability, and longitudinal associations. Annu. Rev. Dev. Psychol. 1, 251–275. 10.1146/annurev-devpsych-121318-085114

[B27] Hollich G. J. Hirsh-Pasek K. Golinkoff R. M. Brand R. J. Brown E. Chung H. L.. (2000). Breaking the language barrier: an emergentist coalition model for the origins of word learning. Monogr. Soc. Res. Child Dev. 65, 1–135. 10.1111/1540-5834.00092 12467096

[B28] Horvath S. Arunachalam S. (2019). Optimal contexts for verb learning. Perspect. ASHA Spec. Interest Groups 4, 1239–1249. 10.1044/2019_PERSP-19-00088 37304204 PMC10256239

[B29] Horvath S. Kueser J. Kelly J. Borovsky A. (2021). Difference or delay? Syntax, semantics, and verb vocabulary development in typically developing and late-talking toddlers. Lang. Learn. Dev. 18, 1–25. 10.1080/15475441.2021.1977645 35664680 PMC9159542

[B30] Lansink J. M. Mintz S. Richards J. E. (2000). The distribution of infant attention during object examination. Dev. Sci. 3, 163–170. 10.1111/1467-7687.00109

[B31] Lee Y. Schumacher E. H. (2024). Cognitive flexibility in and out of the laboratory: task switching, sustained attention, and mind wandering. Curr. Opin. Behav. Sci. 59:101434. 10.1016/j.cobeha.2024.101434

[B32] Maital S. L. Dromi E. Sagi A. Bornstein M. H. (2000). The Hebrew communicative development inventory: language specific properties and cross-linguistic generalizations. J. Child Lang. 27, 43–67. 10.1017/S0305000999004006 10740967

[B33] McGillion M. Herbert J. S. Pine J. Vihman M. dePaolis R. Keren-Portnoy T.. (2017). What paves the way to conventional language? The predictive value of babble, pointing, and socioeconomic status. Child Dev. 88, 156–166. 10.1111/cdev.12671 27859008

[B34] Mesman J. van IJzendoorn M. H. Bakermans-Kranenburg M. J. (2009). The many faces of the still-face paradigm: a review and meta-analysis. Dev. Rev. 29, 120–162. 10.1016/j.dr.2009.02.001

[B35] Morales M. Mundy P. Delgado C. E. F. Yale M. Messinger D. Neal R.. (2000). Responding to joint attention across the 6- through 24-month age period and early language acquisition. J. Appl. Dev. Psychol. 21, 283–298. 10.1016/S0193-3973(99)00040-4

[B36] Moyano S. Rico-Picó J. Conejero Á. Hoyo Á. Ballesteros-Duperón M. de los Á.. (2023). Influence of the environment on the early development of attentional control. Infant Behav. Dev. 71:101842. 10.1016/j.infbeh.2023.101842 37187034

[B37] Provenzi L. Giusti L. Montirosso R. (2016). Do infants exhibit significant cortisol reactivity to the face-to-face still-face paradigm? A narrative review and meta-analysis. Dev. Rev. 42, 34–55. 10.1016/j.dr.2016.07.001

[B38] Rantalainen K. Paavola-Ruotsalainen L. Kunnari S. (2021). Maternal responsiveness and directiveness in speech to 2-year-olds: relationships with children's concurrent and later vocabulary. First Lang. 10.1177/01427237211049585

[B39] Raz H. K. Abney D. H. Crandall D. Yu C. Smith L. B. (2019). “How do infants start learning object names in a sea of clutter?” in Annual Conference of the Cognitive Science Society. Cognitive Science Society (U.S.). Conference 2019, 521–526. 33634271 PMC7903936

[B40] Reilly S. Cook F. Bavin E. L. Bretherton L. Cahir P. Eadie P.. (2018). Cohort profile: the early language in victoria study (ELVS). Int. J. Epidemiol. 47, 11–20. 10.1093/ije/dyx079 29040559

[B41] Reynolds G. D. Romano A. C. (2016). The development of attention systems and working memory in infancy. Front. Syst. Neurosci. 10:15. 10.3389/fnsys.2016.00015 26973473 PMC4776056

[B42] Roberts M. Y. Kaiser A. P. (2015). Early intervention for toddlers with language delays: a randomized controlled trial. Pediatrics 135, 686–693. 10.1542/peds.2014-2134 25733749 PMC4379460

[B43] Salley B. Panneton R. K. Colombo J. (2013). Separable attentional predictors of language outcome. Infancy 18, 462–489. 10.1111/j.1532-7078.2012.00138.x 25342932 PMC4204017

[B44] Stephenson L. J. Edwards S. G. Bayliss A. P. (2021). From gaze perception to social cognition: the shared-attention system. Perspect. Psychol. Sci. 16, 553–576. 10.1177/1745691620953773 33567223 PMC8114330

[B45] Suanda S. H. Smith L. B. Yu C. (2016). The multisensory nature of verbal discourse in parent–toddler interactions. Dev. Neuropsychol. 41, 324–341. 10.1080/87565641.2016.1256403 28128992 PMC7263485

[B46] Tang Y. Gonzalez M. R. Deák G. O. (2024). The slow emergence of gaze- and point-following: a longitudinal study of infants from 4 to 12 months. Dev. Sci. 27:e13457. 10.1111/desc.13457 37941084

[B47] Tardif T. Gelman S. A. Xu F. (1999). Putting the “Noun Bias” in context: a comparison of English and Mandarin. Child Dev. 70, 620–635. 10.1111/1467-8624.00045

[B48] Testa K. McNew M. E. Todd J. T. Eschman B. Bahrick L. E. (2023). Infant distractibility from social events mediates the relation between maternal responsiveness and infant language outcomes. Infant Behav. Dev. 71:101840. 10.1016/j.infbeh.2023.101840 37210883 PMC10512971

[B49] Thelen E. (1995). Motor development: a new synthesis. Am. Psychol. 50, 79–95. 10.1037/0003-066X.50.2.79 7879990

[B50] Tomasello M. Todd J. (1983). Joint attention and lexical acquisition style. First Lang. 4, 197–211. 10.1177/014272378300401202

[B51] Wass S. V. (2015). Applying cognitive training to target executive functions during early development. Child Neuropsychol. 21, 150–166. 10.1080/09297049.2014.882888 24511910 PMC4270409

[B52] Yu C. Suanda S. H. Smith L. B. (2019). Infant sustained attention but not joint attention to objects at 9 months predicts vocabulary at 12 and 15 months. Dev. Sci. 22:e12735. 10.1111/desc.12735 30255968 PMC6918481

[B53] Yurovsky D. Smith L. B. Yu C. (2013). Statistical word learning at scale: the baby's view is better. Dev. Sci. 16, 959–966. 10.1111/desc.12036 24118720 PMC4443688

