# Supplementary material for: Joint attention and exogenous attention allocation during mother-infant interaction at 12 months associate with 24-month vocabulary composition
Source: Front Psychol. 2025 Apr 17;16:1516587. doi: 10.3389/fpsyg.2025.1516587 (PMC12044670; doi:10.3389/fpsyg.2025.1516587)
Supplement: Supplementary file 1 [file Table_1.docx]

**Supplementary Materials – Joint attention and exogenous attention allocation during mother-infant interaction at 12 months associate with 24-month vocabulary composition**

**S1 - Benjamini-Hochberg procedure for multiple comparison correction**

|  |  | **EAA** | **SAM** | **JAI** |
| --- | --- | --- | --- | --- |
| **CDI prod** | **Pearson's r** | -0.323 | 0.270 | -0.042 |
|  | **p-value** | 0.028 | 0.069 | 0.781 |
| **Nouns %** | **Pearson's r** | 0.169 | -0.143 | 0.045 |
|  | **p-value** | 0.261 | 0.343 | 0.766 |
| **Soc %** | **Pearson's r** | 0.129 | -0.079 | -0.024 |
|  | **p-value** | 0.392 | 0.600 | 0.876 |
| **Pred %** | **Pearson's r** | -0.342 | 0.217 | -0.019 |
|  | **p-value** | 0.020 | 0.147 | 0.898 |
| **Closed %** | **Pearson's r** | -0.172 | 0.187 | -0.021 |
|  | **p-value** | 0.254 | 0.213 | 0.888 |

| pvalue | pvalue_ranks | Benjamini-Hochberg |
| --- | --- | --- |
| 0.020 | **1** | **0.01666667** |
| 0.028 | 2 | 0.03333333 |
| 0.069 | 3 | 0.05 |
| 0.147 | 4 | 0.06666667 |
| 0.213 | 5 | 0.08333333 |
| 0.254 | 6 | 0.1 |
| 0.261 | 7 | 0.11666667 |
| 0.343 | 8 | 0.13333333 |
| 0.392 | 9 | 0.15 |
| 0.600 | 10 | 0.16666667 |
| 0.766 | 11 | 0.18333333 |
| 0.781 | 12 | 0.2 |
| 0.876 | 13 | 0.21666667 |
| 0.888 | 14 | 0.23333333 |
| 0.898 | 15 | 0.25 |

| **S2 – Overall Correlation Matrix** | | | | | | | | | |
| --- | --- | --- | --- | --- | --- | --- | --- | --- | --- |
|  |  | **EAA** | **SAM** | **JAI** | **CDI prod** | **Nouns %** | **Soc %** | **Pred %** | **Closed %** |
| **EAA** | **Pearson's r** | — |  |  |  |  |  |  |  |
|  | **p-value** | — |  |  |  |  |  |  |  |
| **SAM** | **Pearson's r** | -0.635 | — |  |  |  |  |  |  |
|  | **p-value** | <.001 | — |  |  |  |  |  |  |
| **JAI** | **Pearson's r** | 0.488 | -0.110 | — |  |  |  |  |  |
|  | **p-value** | <.001 | 0.465 | — |  |  |  |  |  |
| **CDI prod** | **Pearson's r** | -0.323 | 0.270 | -0.042 | — |  |  |  |  |
|  | **p-value** | 0.028 | 0.069 | 0.781 | — |  |  |  |  |
| **Nouns %** | **Pearson's r** | 0.169 | -0.143 | 0.045 | 0.350 | — |  |  |  |
|  | **p-value** | 0.261 | 0.343 | 0.766 | 0.017 | — |  |  |  |
| **Soc %** | **Pearson's r** | 0.129 | -0.079 | -0.024 | -0.812 | -0.756 | — |  |  |
|  | **p-value** | 0.392 | 0.600 | 0.876 | <.001 | <.001 | — |  |  |
| **Pred %** | **Pearson's r** | -0.342 | 0.217 | -0.019 | 0.878 | 0.165 | -0.737 | — |  |
|  | **p-value** | 0.020 | 0.147 | 0.898 | <.001 | 0.274 | <.001 | — |  |
| **Closed %** | **Pearson's r** | -0.172 | 0.187 | -0.021 | -0.303 | -0.867 | 0.608 | -0.229 | — |
|  | **p-value** | 0.254 | 0.213 | 0.888 | 0.041 | <.001 | <.001 | 0.125 | — |
